# Cytotoxic effects of extracts and isolated compounds from *Ifloga spicata* (forssk.) sch. bip against HepG-2 cancer cell line: Supported by ADMET analysis and molecular docking

**DOI:** 10.3389/fphar.2022.986456

**Published:** 2022-09-09

**Authors:** Sajid Hussain, He Liufang, Syed Majid Shah, Fawad Ali, Saeed Ahmad Khan, Fawad Ali Shah, Jing Bo Li, Shupeng Li

**Affiliations:** ^1^ Department of Pharmacy, Kohat University of Science and Technology, Kohat, Pakistan; ^2^ Pediatrics Department, Shenzhen University General Hospital, Shenzhen University, Shenzhen, China; ^3^ Riphah Institute of Pharmaceutical Sciences, Riphah International University, Islamabad, Pakistan; ^4^ Shenzhen University Clinical Research Center for Neurological Diseases, Health Management Center, Shenzhen University General Hospital, Shenzhen University Clinical Medical Academy, Shenzhen University, Shenzhen, China; ^5^ State Key Laboratory of Oncogenomics, School of Chemical Biology and Biotechnology, Shenzhen Graduate School, Peking University, Shenzhen, China

**Keywords:** Ifloga spicata, brine shrimp lethality, MTT assay, tyrosine kinase, molecular docking

## Abstract

The purpose of this study was to determine the anticancer potential of *Ifloga spicata* (*I. spicata*) against HepG-2 cell line. To assess *I. spicata* cytoxicity, brine shrimp lethality and MTT assays were performed. In the brine shrimp bioassay, the ethyl acetate fraction had a significant impact with an IC_50_ of 10 μg/ml. The ethyl acetate and chloroform fractions inhibited HepG-2 cell line effectively (IC_50_ values 5.54 and 6.52 μg/ml, respectively). The isolated compound, heptadecyl benzoate inhibited growth significantly (IC_50_, 8.92 μg/ml) while methyl dihydroxybenzoate had modest activity (25.66 μg/ml) against the cell line. Both compounds displayed acceptable pharmacokinetic parameters in the ADME study. In the docking study, the methyl dihydroxybenzoate was involved in two hydrogen bonds with two different residues Thr830 and Asp831. The heptadecyl benzoate carbonyl oxygen exhibited a single hydrogen bond with Lys692. Both showed good interactions with the active site of the (EGFR) tyrosine kinase. Our findings suggest that *I. spicata* might be a viable source of anticancer natural agents. This discovery raises the prospect of the future development of a new medication for the treatment of liver cancer.

## Introduction

Cancer is the world’s second greatest cause of mortality, after cardiovascular illnesses, with approximately 10 million deaths in 2020 (Sung et al., 2021). Despite recent advancements in cancer therapy, this figure is anticipated to double in the next 10 years (N’guessan et al., 2021; Ferlay et al., 2019). Liver cancer is the principal reason of death associated with cancer across the globe. Liver cancer is the 5th and 9th most common cancer among men and females, respectively, and the 2nd cause of mortality across the globe (Ferlay et al., 2015). According to the published data, the annual number of reported cases of liver cancer is 600,000–800,000, accounting for about 5.6% of all cancers. It was also expected that the number of cases will be about 100,0000 by 2030 (Alqahtani et al., 2019).

Medicinal herbs have a long history of being used to cure a variety of illnesses, including cancer (Khan et al., 2019; Xiao et al., 2020). More than 60% of cancer medications, such as doxorubicin and bleomycin, are derived from natural sources, such as plants and microbes (Cragg and Newman, 2005). Many plant-derived natural agents have potential value as chemotherapeutic agents (Pezzuto, 1997). Several useful anticancer agents in clinical use are obtained from plants like vinblastine and vincristine obtained from *C. roseus,* etoposide from lignans of Podophyllum spp, paclitaxel isolated from the bark of the Pacific yew and camptothecin derivatives, like topotecan, isolated from *Camptotheca acuminate* (Newman et al., 2003; Basili and Moro, 2009).

Tyrosine kinases (TKs) are useful in the development of cancer-targeting drugs (Paul and Mukhopadhyay, 2004). For both external and internal stimuli, TKs are required for several biological operations like apoptosis, growth and migration, differentiation, and metabolism (Vlahovic and Crawford, 2003). TKs are enzymes that start the phosphorylation process for certain tyrosine residues with the help of ATP. Following biosynthesis, protein covalent and enzymatic modification is a crucial aspect of maintaining normal cellular communication and homeostasis (Hussain et al., 2019).

*I. spicata* is an annual herb used to treat skin problems and heart disorders (Abouri et al., 2012)*. I. spicata* has been shown to have anti-leishmanial properties. Furthermore, the anticancer activity of *I. spicata* against lung cancer has been scientifically proven. (Shah et al., 2019a; Shah et al., 2019b). The current study aimed to evaluate the pharmacokinetic profile, tyrosine kinase receptor sensitivity and the anticancer effects of fractions and isolated compounds of *I. spicata* using brine shrimp lethality and MTT assays*.*

## Materials and methods

### Chemicals and equipment

The extraction solvents (ethyl acetate, chloroform*, n-hexane*, and methanol) were acquired from Daejung Korea. Sigma Chemicals Co. MTT dye and dimethyl sulfoxide were obtained from Sigma Chemicals Co. (St. Louis, United States). The standards etoposide (Cas number 33419-42-0) and doxorubicin (Cas number 25316-40-9) were purchased from Merck. Silica gel 60 F254 cards was purchased from Merck. Silica. gel, 70–230 mesh, obtained from Scharlau, Spain. ELISA reader (ELx800 BioTek) and Rota vapour 210 were used for recording the absorbance and concentrating the fractions respectively.

### Preparation of crude extract and its subsequent fractions

The *I. spicata* was collected from District, Karak, Khyber Pakhtunkhwa, Pakistan in the month of April 2016. The plant material was identified by taxonomist Dr. Waheed Murad, Department of Botany, Kohat university of science and technology, Kohat. A voucher specimen with catalog no KUH-1002 was deposited in the herbarium of the Department of Botany, Kohat University of Science and Technology, Kohat. The plant material was shade-dried and crushed into coarse powder after being collected. The plant’s coarse powder was macerated in methanol for 15 days after being shade dried. By using rotary evaporator, the filtrate was concentrated to get methanol extract. The methanolic extract of 500 g was dispersed in distilled water and sequentially extracted with organic solvents. *n-hexane*, chloroform, and ethyl acetate to get polarity-based fractions. All fractions were kept in the refrigerator until they were utilized in experiments.

### Isolation scheme

The most bioactive ethyl acetate-soluble fraction was analyzed using column chromatography with a mobile phase of *n-hexane*/ethyl acetate and a stationary phase of flash silica gel. Column chromatography was performed with the help of a column (4.6 mm internal diameter and 30 cm in length). for the most bioactive ethyl acetate-soluble fraction using a mixture of n*-hexane*/ethyl acetate [Daejung CAS No.110-54-3, (Daejung CAS No.141-78-6)] as the mobile phase, and flash silica gel (230–400 mesh) as the stationary phase and eluted two sub fractions. The two eluted sub fractions were subject to repeated column chromatography using a pencil column (3.9 × 15). Methyl dihydroxybenzoate (15 mg) was obtained from fraction 1 using mobile phase n-hexane/ethyl acetate in the ratio 90:10, and Heptadecyl benzoate (12 mg) from fraction 2 using n-hexane/ethyl acetate in the ratio 94:6. Aluminum sheets precoated with silica gel 60 F2s4 (20 cm, 0.2 mm thick; E-Merck) were used for TLC and visualization of the TLC plates was carried out under UV at 254 and 366 nm and also by spraying ceric sulphate reagent with heating. Various spectroscopic methods, such as ^1^H and ^13^C techniques, including homonuclear (COSY) and heteronuclear correlation tests (HSQC and HMBC), as well as a literature survey, were used to confirm the structure of the compounds (http://www.mdpi.com/2227-9717/7/4/208/s1) as previously published (Shah et al., 2019c).

### Brine shrimp lethality assay

This assay was used to assess the toxicity of *I. spicata* crude extract and its subsequent fractions to brine shrimp. Brine shrimp eggs were hatched in a beaker containing seawater. Plant samples were dissolved in DMSO (Meyer et al., 1982; Mohtasheem et al., 2001). Plant extracts at a concentration range of 10–1000 μg/ml were used in the lethality assay. 30 live shrimps were added to each container containing 5 ml of simulated seawater (composition: sea salt 38 g/L of DW having pH 7.4). 100 μL of each plant extract was added to containers and incubated for 24 h. After incubation, the shrimps that survived were counted in each container. As a standard, etoposide (1000, 100. 10 μg/ml) was utilized. The negative control was DMSO. The percentage mortality of plant samples was calculated. The IC_50_ value for each extract of *I. spicata* was determined.

### Cell culture

Cancer cells (HepG-2) were obtained from the National Centre of Excellence in Molecular Biology at the University of Punjab in Pakistan. Cancer and normal cells (Vero) were nurtured in DMEM. The cultured cells were preserved until they were utilized in research.

### Cytotoxicity assay

The cytotoxicity of plant fractions and the compounds was determined by using a 3-(4,5-Dimethylthiazol)-diphenyl tetrazolium bromide (MTT) assay (Mosmann, 1983; Khorrami et al., 2018). The normal Vero and HepG2 cell lines were cultured in DMEM containing antibiotics (streptomycin/penicillin) and nutrients 10% fetal bovine serum. The normal and cancer cells were incubated in a CO_2_ incubator for 24 h at 37°C. Both the cell lines were treated with different concentrations in a range of 6.25–200 μg/ml with the extracts and compounds and incubated for a further 1 day. Doxorubicin in a concentration range of 6.25–200 μg/ml and under same experimental conditions was used as standard. DMSO was used as negative control in this MTT assay. A 100 μL of MTT reagent (5 mg/ml in phosphate buffer saline) is added to each well and incubated for 4 h at 37°C in a 5% CO_2_ atmosphere. Absorbance was measured at 570 nm using an ELISA reader. By using the following formula, the percentage of cytotoxicity was determined:

Cell viability % = absorbance of treated cells/absorbance of control cells × 100.

The experiments were done in triplicates. The IC_50_ values were calculated for each plant sample.

### ADMET analysis

Swiss-ADME (http://www.swissadme.ch/index.php) tool was used for ADMET analysis (Absorption, distribution, metabolism, excretion and Toxicity) (DeLano, 2002). The ADMET analysis is considered too important for a molecule to be considered a drug candidate (Hassan et al., 2022). The pharmacokinetic scores of compounds were measured using the online database pkCSM (http://biosig.unimelb.edu.au/pkcsm/prediction **).**

### Molecular docking study

The MOE-2016 software was used for the docking study of compounds 1 and 2 against the target enzyme, Epidermal Growth Factor Receptor (EGFR) tyrosine kinase domain (EC 2.7.10.1) having PDB ID: 1M17. The 3D structures of compounds and target enzymes were built and the energy of compounds was minimized up to 0.05 gradient using MMFF 94s forcefield (Mphahlele et al., 2017).

By using the Triangular Matching docking method, 10 different conformations for each compound were generated based on the binding of compounds with the active sites of receptor and binding energies were determined. The molecular interactions of ligand-protein complexes were noted. By using LigPlot 3D images were drawn.

## Results

### Brine shrimp lethality bioassay

**FIGURE 1:**
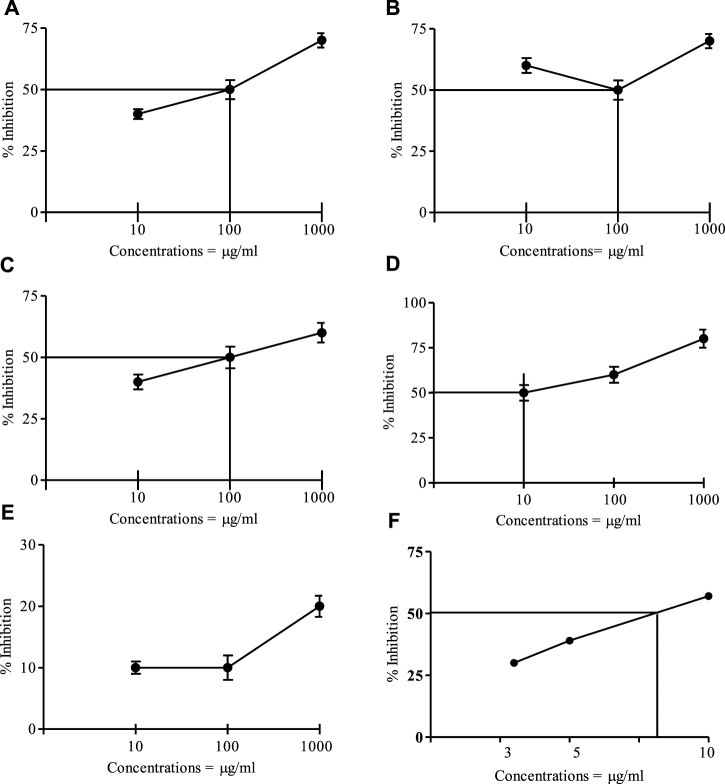
Brine shrimp cytotoxic effect of the crude and fractions of *I. spicata* at various Concentrations. **(A)** = Crude drug, **(B)** = Hexane fraction; **(C)** = Chloroform fraction; **(D)** = Ethyl acetate fraction; **(E)** = Water fraction; **(F)** = Etoposide, IC_50_ = 7.35 μg/ml. Data are mean ±SEM of three independent readings).

Figure 1 represents the cytotoxicity of *I. spicata* crude and resulting fractions. The ethyl acetate component of the plant displayed a promising impact with an IC_50_ of 10 μg/ml. When compared to the control medication, etoposide, the mortality effects of crude extract, *n-hexane*, and chloro-form fractions were modest. The aqueous fraction was inactive.

### *In vitro* cytotoxicity

An MTT assay was used to assess the cytotoxic activity of *I. spicata* extracts and isolated components. Table 1 summarizes the findings of the experiment. The ethyl acetate and chloroform fractions were regarded as the most potent fractions, with IC_50_ values of 5.54 and 6.52 μg/ml, respectively. The results indicated that the crude extract and *n-hexane* inhibited the cancer cell line effectively. In comparison to other plant fractions, the aqueous fraction’s anticancer efficacy was modest. A significant inhibition of growth was recorded for heptadecyl benzoate with an IC_50_ value of 8.92 μg/ml. The plant extracts and compounds did show any inhibitory effect against the normal Vero cell line. The methyl dihydroxybenzoate had moderate anticancer activity.

**TABLE 1:** *I. spicata* fractions and compounds cytotoxicity against HepG-2 cell line.

Plant samples	Concentration (μg/ml)	Percent inhibition against HepG-2 cell lin	IC_50_ (μg/ml) against HepG-2 cell line
Crude extract	200	86.6 ± 1.1	17.82
	100	76.7 ± 1,2	
	50	65.2 ± 0.9	
	25	57.7 ± 0.6	
	12.5	44.6 ± 0.5	
	6.25	31.7 ± 0.8	
*n-Hexane*	200	77.7 ± 0.5	27.62
	100	72.4 ± 0.3	
	50	60.3 ± 0.3	
	25	50.8 ± 0.2	
	12.5	35.9 ± 0.2	
	6.25	25.5 ± 0.2	
Chloroform	200	81.5 ± 1.0	6.52
	100	78.8 ± 0.5	
	50	71.6 ± 0.9	
	25	69.6 ± 0.6	
	12.5	60.3 ± 0.7	
	6.25	44.5 ± 1.2	
Ethyl acetate	200	91.7 ± 0.5	5.54
	100	84.5 ± 0.6	
	50	75.7 ± 0.9	
	25	70.6 ± 0.2	
	12.5	61.7 ± 0.9	
	6.25	52.7 ± 0.6	
Aqueous	200	60.7 ± 0.4	71.81
	100	54.5 ± 0.2	
	50	46.7 ± 0.6	
	25	38.7 ± 0.9	
	12.5	30.9 ± 0.5	
	6.25	17.9 ± 0.4	
Methyl dihydroxybenzoate	200	78.6 ± 1.1	25.66
	100	69.9 ± 0.9	
	50	59.6 ± 0.7	
	25	49.8 ± 0.9	
	12.5	42.6 ± 0.4	
	6.25	26.5 ± 1.1	
Heptadecyl benzoate	200	89.6 ± 0.9	8.92
	100	78.5 ±0.3	
	50	69.5 ± 0,7	
	25	64.4 ± 0.9	
	12.5	53.4 ± 0.6	
	6.25	47.7 ± 0.3	
Doxorubicin	200	90.1 ± 0.2	1.25
	100	84.4 ± 0.4	
	50	79.6 ± 0.3	
	25	74.8 ± 0.4	
	12.5	70.3 ± 0.3	
DMSO	0 μg/ml	NA	NA

Data are represented as mean ± S.E.M of three independent readings (*n* = 3). The reference standard, Doxorubicin, IC_50_ = 1.25 μg/ml. NA, Not active.

### ADMET analysis (pharmacokinetic and toxicological properties)

The physicochemical properties of the compounds are mentioned in Table 2. The physicochemical features were investigated and classified into six broad groups based on their oral bioavailability ranges. Both compounds’ results are within the acceptable ranges.

**TABLE 2:** Physicochemical and lipophilicity properties of methyl dihydroxybenzoate and heptadecyl benzoate.

Properties	Parameters	Methyl dihydroxybenzoate	Heptadecyl benzoate
Physicochemical properties	MW^a^ (g/mol)	152.15	360.57
	Rotatable bonds	2	18
	HBA^b^	3	2
	HBD^c^	1	0
	Fraction Csp3	0.12	0.71
	TPSA^d^	46.53	26.30
Lipophilicity Log P_o/w_	iLOGP	1.63	5.68
	XLOGP3	1.96	10.58
	MLOGP	1.32	5.98
	Consensus	1.46	7.60

^a^ Molecular weight

^b^ Hydrogen bond acceptor

^c^ Hydrogen bond donor

^d^ Typological polar surface area

**FIGURE 2:**
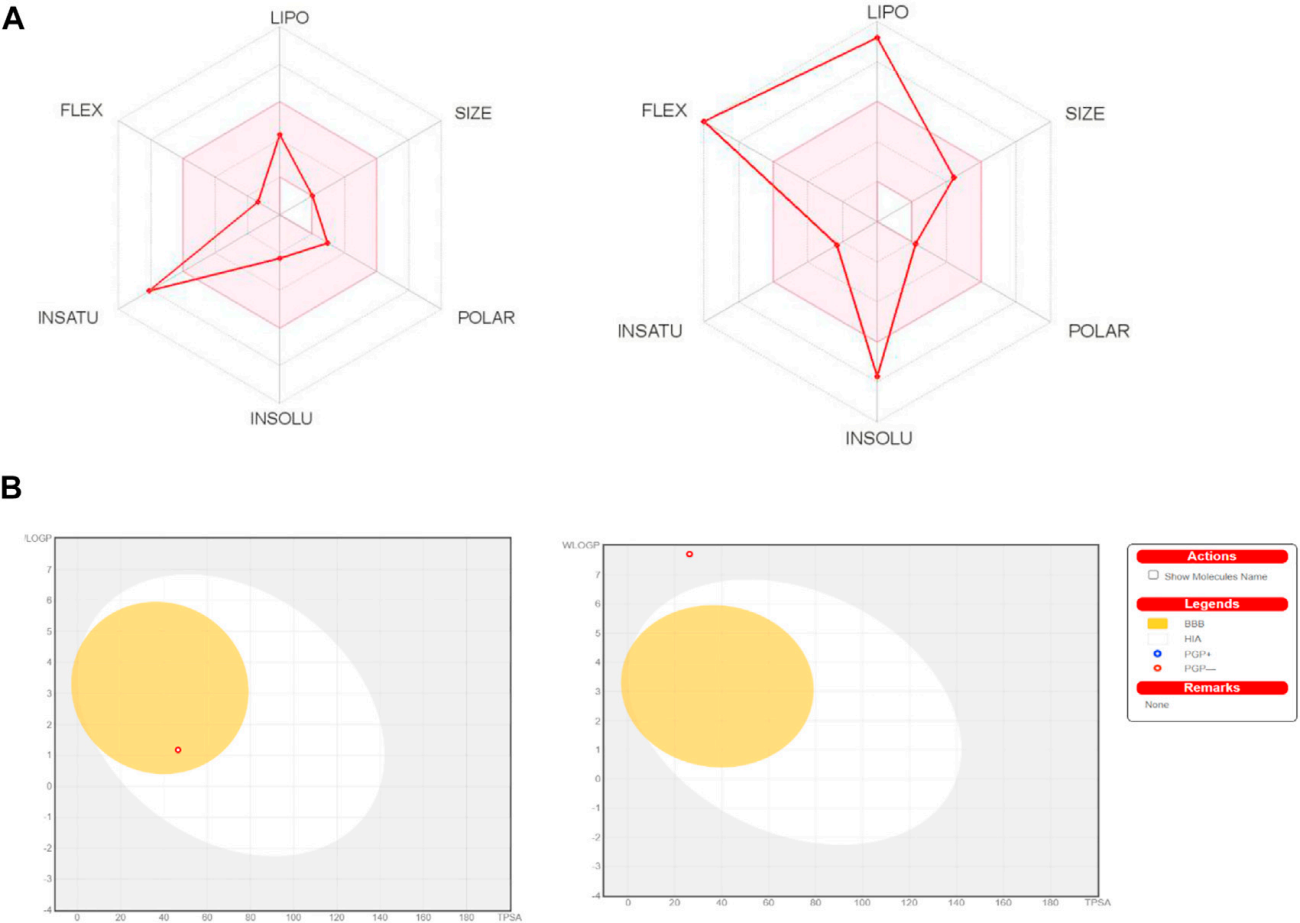
**(A)** Bioavailability radar chart for methyl dihydroxybenzoate and heptadecyl benzoate respectively. **(B)** Predicted BOILED-Egg plot from *swiss ADME* online web tool for methyl dihydroxybenzoate and heptadecyl benzoate respectively.

The chemical absorption from the gastrointestinal absorption region (GI^a^) and the blood brain barrier (BBB) permeation are shown in the BOILED-EGG graph. Methyl dihydroxybenzoate has higher oral bioavailability, as seen in Figure 2A of the Bioavailability Radar Chart. Figure 2B shows that methyl dihydroxybenzoate is in the white zone, while heptadecyl benzoate is in the grey zone. Methyl dihydroxybenzoate had a high GI^a^ and BBB permeability (Table 3), but heptadecyl benzoate had a low GI^a^ and no BBB permeation. Methyl dihydroxybenzoate inhibits CYP1A2, whereas the heptadecyl benzoate inhibits two isoforms, CYP1A2 and CYP2C19, resulting in considerable pharmacological interaction with the substrates of these two isoforms. The clearance value for methyl dihydroxybenzoate was insufficient. Both compounds appear to be non-substrates of Organic Cation Transporter 2 (OCT2), and their interaction with an OCT2 inhibitor promotes unfavorable interactions Table 3.

**TABLE 3:** Predicted pharmacokinetics parameters of methyl dihydroxybenzoate and heptadecyl benzoate.

Properties	Parameters	Methyl dihydroxybenzoate	Heptadecyl benzoate
Absorption	Water solubility (log S(ESOL))	−1.695	−7.329
	GI^a^ (gastrointestinal absorption)	High	Low
	Skin Permeability cm/s	−2.342	−2.676
	P-glycoprotein substrate	No	No
Distribution	VDss (volume of distribution at steady state, (human)	−0.165	0.677
	Fraction unbound (human)	0.448	0
	BBB permeability	Yes	No
	CNS permeability (Log PS)	−2.072	−1.152
	CYP1A2 inhibitor	Yes	Yes
	CYP2C19 inhibitor	No	Yes
	CYP2C9 inhibitor	No	No
	CYP2D6 inhibitor	No	No
	CYP3A4 inhibitior	No	No
Excretion	Total Clearance (log ml/min/kg)	0.73	1.95
	Renal OCT2 substrate	No	No

Furthermore, the online database pKCSM was used to examine the toxicity profile of methyl dihydroxybenzoate and heptadecyl benzoate (Table 4). Methyl dihydroxybenzoate had a greater maximum tolerated dose (MTD) than heptadecyl benzoate. The MTD is a unit of measurement for a chemical’s toxic dose threshold in humans. Both compounds did not reduce hERG I, however, only heptadecyl benzoate inhibited hERG II, according to our findings. Skin hypersensitive reaction is a possible adverse effect of the chemical, and heptadecyl benzoate has demonstrated skin allergies. Furthermore, both substances have a low score level for calculating fatal dose (LD_50_), i.e., 2.793 mg/kg and 3.362 mg/kg, and were classified as class 1.

**TABLE 4:** Toxicity profile of methyl dihydroxybenzoate and heptadecyl benzoate.

Parameters	Methyl dihydroxybenzoate	Heptadecyl benzoate
AMES toxicity	No	No
Max. tolerated dose (human) (log mg/kg/day)	1.215	0.796
hERG I inhibitor	No	No
hERG II inhibitor	No	Yes
Oral Rat Toxicity (LD50) (mg/kg)	2.793	3.362
Hepatotoxicity	No	No
Skin Sensitization	No	Yes

### Compound-target enzyme interactions

**FIGURE 3:**
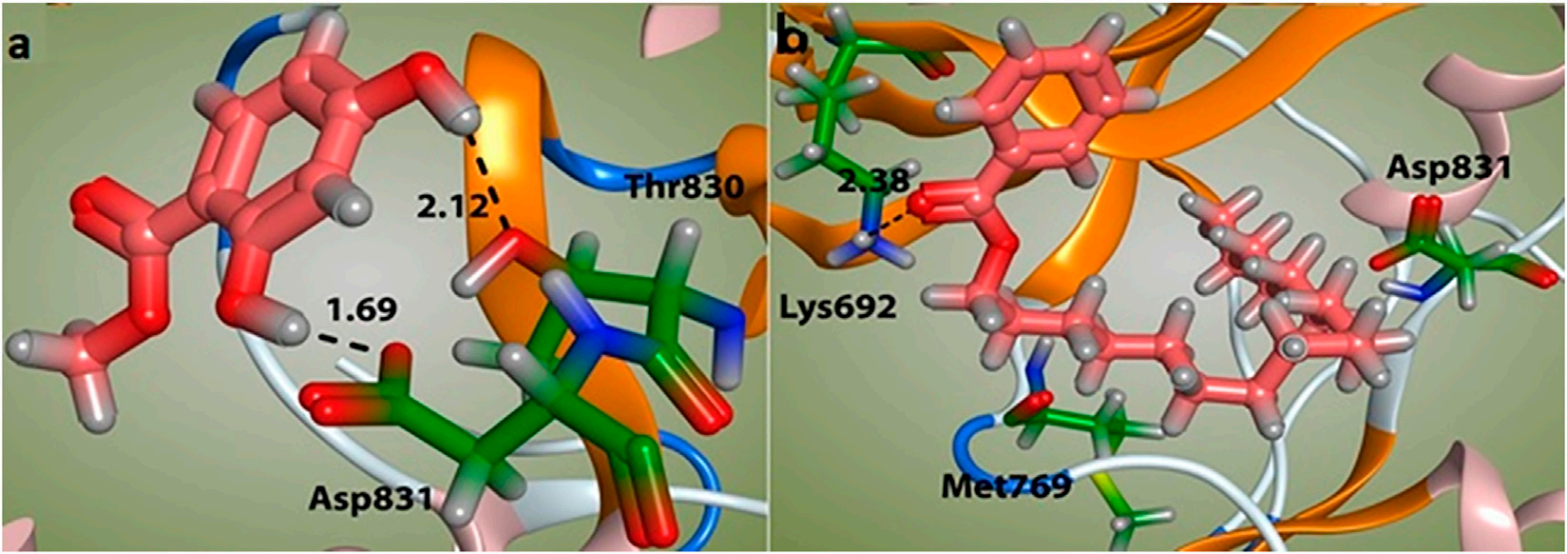
Binding mode of compounds methyl dihydroxybenzoate **(A)** and heptadecyl benzoate **(B)** with the target enzyme, (EGFR) tyrosine kinase. The ligands are shown as pink and residues as green.

The docking study was conducted to investigate the binding poses of isolated compounds from *I. Spicta* in the active site of (EGFR) tyrosine kinase. The compounds fit best in the active site and have favorable interactions with the active site residues. The methyl dihydroxybenzoate was involved in two hydrogen bonds with two different residues Thr830 and Asp831. The heptadecyl benzoate carbonyl oxygen shows a single hydrogen bond with Lys692. The interactions shown by the compounds may be due to the presence of hydrogen bond donor and hydrogen bond acceptor groups. Figure 3 shows compound 3D interactions with (EGFR) tyrosine kinase active site residues. The drug-likeness properties, binding energies, docking score and interaction detail of compounds are summarized in Tables 5, 6.

**TABLE 5:** The structure and Lipinski’s properties of ligands.

S.No	Compound	Structure	Properties
1	Methyl 2,4-dihydroxybenzoate	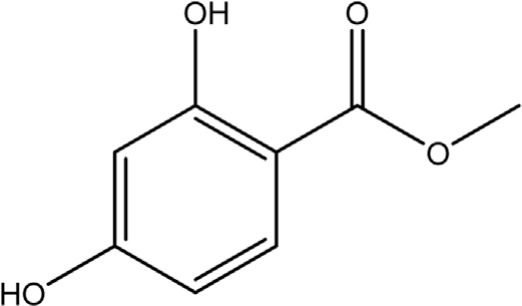	MW 168.15 g/mol, LogP 0.92, Don 2, Acc 3
2	Heptadecyl benzoate	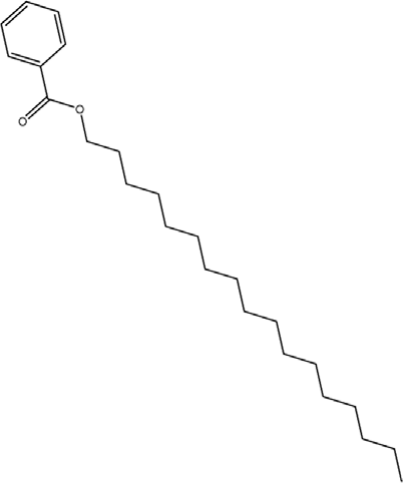	MW 360.58 g/mol, LogP 9.49, Don 0, Acc 1

**TABLE 6:** Docking score, binding energies and Predicted interactions of compounds.

S.No	Docking score	Binding energy	Binding affinity	Interaction of compounds with the receptor
1	−4.759	−26.51	−5.07	Ligand Receptor Interaction Distance
				O 10 OG1 THR 830 H-donor 2.12
				O 12 OD2 ASP 831 H-donor 1.69
2	−5.801	−33.45	−6.4	Ligand Receptor Interaction Distance
				O 63 NZ LYS 692 H-acceptor 2.38

## Discussion

The brine shrimp lethality test is a commonly used bioassay for preliminary screening in the discovery of anticancer medicines (Ullah et al., 2013). The ethyl acetate exhibited a marked effect with IC_50_ of 10 μg/ml. In comparison to standard etoposide, crude extract, *n-hexane*, and chloroform fractions had a mild impact. For the aqueous fraction, no impact was seen. It demonstrates that cytotoxic plant components are higher concentrated in the ethyl acetate fraction. The cytotoxic effect of the plant organic fractions might be due to the active constituents present in the plant extracts Therefore, the cytotoxic effects of the tested samples indicate that they can be selected for further study against human cancer cell lines (Manilal et al., 2009). It is thus stated that plant *I. spicata* possess cytotoxic effects which are in accordance with the findings obtained earlier and can be used as a source of potential anticancer agents (Shah et al., 2015; Naher et al., 2019).

Polyphenols are a kind of phytochemical that has lately gained popularity due to their potential to prevent cancer cell development. Polyphenols have been shown to cause cell cycle arrest in malignancies such as gastric cancer, hepatoma, endometrial cancer, nasopharyngeal cancer, prostate cancer, breast cancer, pancreatic cancer, and lymphoma cancer. Resveratrol, a stilbene, and quercetin, a flavonol, are two polyphenols that limit cancer cell proliferation. As a result, their effects have been investigated in a variety of cancers, including breast, prostate, hepatoma, and colon cancer (Kampa et al., 2007). The MTT assay is the most commonly used technique to evaluate cell viability and proliferation (Table 1). The findings of this study are consistent with earlier studies that show similar effects are caused by the presence of various bioactive substances (Kumar and Pandey, 2013; Taiwo et al., 2020). The crude extract and *n-hexane* fractions showed concentration-dependent inhibition of HepG-2 cell line in tested concentration range of 6.25–200 μg/ml. In a previous study the anticancer activity of *I. spicata* against lung cancer has also been reported (Shah et al., 2019a). A new juglone analog, 2-ethoxystypandrone, derived from ethyl acetate extract of the roots of *Polygonum cuspidatum,* has been shown to have anticancer action against hepatocellular carcinoma (Shah et al., 2019c). The activity of aqueous extract was low as compared to other fractions of plant. A significant inhibition of growth was observed for heptadecyl benzoate with an IC_50_ value of 8.92 μg/mL. A moderate activity (IC_50_ = 25.66 μg/ml) was shown by methyl dihydroxybenzoate. In another study glycoalkaloids isolated from *Salanum melongena* demonstrated a significant antiproliferative effect against liver cancer cells (Fekry et al., 2019).

This study uses cytotoxicity, ADMET, and molecular docking approaches to assess the efficacy of extracts and isolated compounds of *I. spicata* as a putative biomolecule therapeutically active against HepG-2 Cancer Cells. The physicochemical features of compounds were studied. The results of both compounds fall within the limits, demonstrating their potential as drug candidates for clinical trials.

The BOILED-EGG graph indicates the compound absorption from the GI^a^ and the BBB permeation. The white area is for GI^a^ showing the compound absorption region, and the yellow zone (yolk) is for the BBB diffusion. While if the compound occurs in the grey geographical zone, it suggests that the compound has neither GI absorption nor it crosses BBB (Daina and Zoete, 2016). GI^a^ and CNS absorption are crucial factors examined for every single drug candidate prior to its access to drug formulation (Chen et al., 2020). The Blood-brain barrier penetration is necessary for the compound to have actions on the central nervous system (Cruz et al., 2018). In Bioavailability radar chart, methyl dihydroxybenzoate showed better oral bioavailability. The methyl dihydroxybenzoate has presented a high GI^a^ with BBB permeability than Heptadecyl benzoate, indicating that heptadecyl benzoate low occurrence for CNS side effects. Both are not P-gp (P-glycoprotein) substrate hence they are not prone to the efflux mechanism of P-gp, as many cancer lines exploited that mechanism as a source of drug resistance (Binkhathlan and Lavasanifar, 2013). Furthermore, the methyl dihydroxybenzoate showed less skin permeation, as more the negative Log Kp valve of the molecule results in less skin permeability (DeLano, 2002).

Furthermore, the most common cytochromes isoforms were analyzed against these compounds as these enzymes have a significant part in drug excretion. Inhibition of these isoforms results in drug interaction (Chen et al., 2020). The heptadecyl benzoate inhibits more isoforms, causing significant drug interaction. Calculation of drug clearance of a compound is critical for a molecule to find steady-state concentrations. The methyl dihydroxybenzoate clearance value was inadequate. Both compounds showed as non-substrate of Organic Cation Transporter 2 (OCT2).

Drug toxicity profiling is necessary before a drug enters into the clinical trials or in the pre-formulation stage [25]. Therefore compounds have been assessed for toxicities i.e., human, oral and rat. The AMES toxicity test normally estimates the mutagenic ability of a compound. In this study, we found that both compounds were characterized as non-Ames risky, representing no carcinogenic ability. The maximum tolerated dose (MTD) for methyl dihydroxybenzoate was higher than heptadecyl benzoate. The MTD is a quantity of a chemical’s harmful dosage threshold in humans. In our findings, hERG I was not suppressed by both compounds but hERG II was inhibited only by heptadecyl benzoate, hERG-encoded potassium channels inhibition consequence in ventricular arrhythmia by QT prolongation (Robertson and Morais-Cabral, 2020). The isolated compounds were also predicted as non-hepatotoxic, therefore considered as a safe drug in terms of drug-induced liver adverse effects (Hoitsma et al., 1991). A probable side effect of a molecule is a skin hypersensitivity reaction, heptadecyl benzoate has shown skin allergies.

The docking analysis was used to identify the binding poses of the isolated compounds in the active site of (EGFR) tyrosine kinase. In docking tests, both agents demonstrated a high binding affinity (Docking score −4.759, binding energy −26.51 and binding affinity −5.07 for methyl dihydroxybenzoate. Docking score −5.801, binding energy −33.45 and binding affinity −6.4 for heptadecyl benzoate). The docking results supported the *in vitro* results against the cancer cells. Our findings are consistent with previous studies (Galal et al., 2014). In our previous study, a novel compound, 3-hydroxyoctyl-5, trans-docosenoate, isolated from the plant, *Anchusa arvensis* has exhibited significant interaction with tyrosine kinase enzyme (Hussain et al., 2019). Therefore, it could be assumed that these two compounds might have anticancer effects through tyrosine kinase. However, it will require further experimental confirmation.

## Conclusion

The current study provides new understanding of the anticancer potential of *I. spicata* against liver cancer. The ethyl acetate fraction had a significant effect in the brine shrimp mortality experiment, with an IC_50_ of 10 μg/mL. A significant effect was exhibited by heptadecyl benzoate. Both compounds exhibited acceptable pharmacokinetic properties except in absorption of heptadecyl benzoate in ADME analysis. Both isolated compounds showed a safer profile in toxicity assessment. Furthermore, both compounds showed marked binding affinity towards (EGFR) tyrosine kinase, supporting the *in-vitro* effects. Our results suggest that *I. spicata* has bioactive compounds with promising anticancer potential. Therefore, it can be further used for the development of drugs for liver cancer treatment.

## Data Availability

The original contributions presented in the study are included in the article/Supplementary Materials, further inquiries can be directed to the corresponding authors.
